# The First COL4A5 Exon 41A Glycine Substitution in a Family With Alport Syndrome

**DOI:** 10.3389/fped.2020.00153

**Published:** 2020-04-09

**Authors:** Fang Wang, Dan Zhao, Jie Ding, Xuejuan Li

**Affiliations:** Department of Pediatrics, Peking University First Hospital, Beijing, China

**Keywords:** Alport syndrome, COL4A5, p. (Gly1264Asp), alternative splices, phenotype

## Abstract

**Background:** X-linked Alport syndrome is caused by mutations in the COL4A5 gene, which encodes the a5(IV) chain. No mutations were detected in COL4A5 exons 41A and 41B.

**Materials and Methods:** A Chinese family with suspected Alport syndrome was enrolled in the present study to establish a precise diagnosis. The proband's father and uncle progressed to end-stage renal failure at different age. The indirect immunofluorescence method was used for analysis of distribution of a1 (IV) and a5 (IV) chains in the epidermal basement membrane from the father of the proband. The entire coding region of COL4A5 mRNA from the proband's father cultured skin fibroblasts was analyzed by using reverse-transcription polymerase chain reaction (RT–PCR) and direct sequencing, and genomic DNA was analyzed by using PCR and direct sequencing. To examine whether the alternatively COL4A5 mRNA transcripts existed in cultured skin fibroblasts, a fragment of COL4A5 cDNA, including exons 41A, 41B, and partial sequences of exons 41 and 42 was analyzed by RT–PCR and GeneScan.

**Results:** Negative a5(IV) chain staining in the epidermal basement membrane was detected in the female proband's father who presented with hematuria, proteinuria, and renal dysfunction. Sequencing analysis demonstrated that the proband's father had a novel variant c.3791G>A [p. (Gly1264Asp)] in COL4A5 exon 41A detected at the mRNA and genomic DNA levels, and the variant segregated with disease in the family. According to the phenotype and American College of Medical Genetics and Genomics guideline, this variant was considered clinically pathogenic. The GeneScan analysis showed three COL4A5 mRNA transcripts expressed in the cultured skin fibroblasts of the proband's father and two normal males, and variation could be seen in the amounts of amplified isoforms.

**Conclusions:** A glycine substitution in COL4A5 exon 41A was identified in a family with intrafamilial heterogeneity of the rate of progression to end-stage renal failure in male patients, which extends the phenotypic and mutational spectrum of X-linked Alport syndrome. In addition, skin tissue has three distinct COL4A5 transcripts with a diversity of expression.

## Introduction

Alport syndrome (AS) is a progressive hereditary nephropathy, characterized by hematuria, proteinuria, and inevitable progression to end-stage renal failure (ESRD). Extrarenal manifestations including hearing loss, ocular lesions, and leiomyomatosis can be observed. Pathogenic variants in the COL4A3, COL4A4, and COL4A5 genes, encoding a3(IV), a4(IV), and a5(IV) chains, respectively, are responsible for AS. Although a molecular diagnosis of this disease has been recommended (1), AS can also be diagnosed even when one patient with glomerular hematuria fulfills one of the following clinical criteria (2, 3): (a) ultrastructural changes in the glomerular basement membrane (GBM) typical of AS; (b) abnormal immunostaining of the a5 (IV) chain in the epidermal basement membrane (EBM); and (c) abnormal immunostaining of the a3 (IV), a4 (IV), and a5 (IV) chains in GBM.

AS can be transmitted as an X-linked, autosomal recessive, autosomal dominant, or digenic trait (1, 4–6), of which X-linked inheritance with mutations in the COL4A5 gene is most common. Until now, the Human Gene Mutation Database (HGMD) contains more than 1,000 mutations in COL4A5, of which glycine substitution is the most frequent type of mutation (2, 7). The studies of genotype-phenotype correlation in X-linked AS reveal that age at onset of ESRD and extrarenal manifestations in male patients are influenced by COL4A5 mutation categories, whereas the variability of disease severity in females is less well-explained by the genotypes (7–10).

Three distinct COL4A5 transcripts with different tissue expression have been identified: transcript variant I containing exons 41A and 41B is present in human kidney, liver, placenta, and spleen tissues; transcript variant II containing exon 41B is present only in human epithelial tissue; and transcript variant III without exons 41A and 41B is present in human kidney, liver, placenta, spleen and epithelial tissues (11, 12). To our knowledge, no variations have been detected in the COL4A5 exons 41A and 41B. Here, we describe a family with a novel COL4A5 missense mutation c.3791G > A [p. (Gly1264Asp)] in exon 41A and demonstrate that three distinct COL4A5 transcripts are present in skin tissue.

## Materials and Methods

### Patient

**Figure 1:**
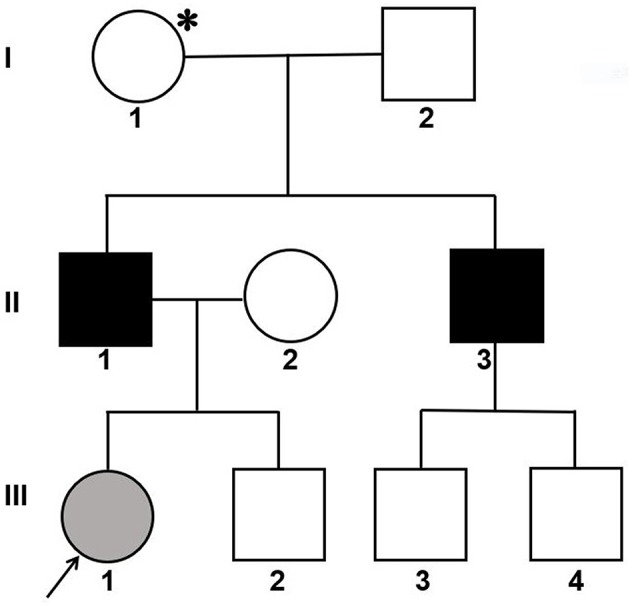
Pedigree of a Han Chinese family with X-linked Alport syndrome. The filled gray circle indicates the individual with hematuria detected by urine microscopy. The filled black squares indicate the individuals with hematuria, proteinuria and renal failure. The asterisk indicates the individual with hematuria detected by dipstick urinalysis. The arrow indicates the proband.

An affected Han Chinese familial pedigree is shown in Figure 1. The female proband (III-1) was found to have isolated glomerular microscopic hematuria at the age of 2.4 years. She is now 14 years of age and has normal blood pressure, microscopic hematuria without proteinuria, and normal renal function. She had a paternal family history of hematuria, proteinuria, and renal dysfunction. Her father (II-1) was coincidentally found to have microscopic hematuria and proteinuria at the age of 24 years. At age 27, his blood pressure remained normal, urinary protein excretion was 2.18 g/24 h, renal function remained normal, and renal biopsy showed focal segmental glomerulosclerosis. Glomerular basement membrane ultrastructural findings were not obtained. At age 37, although treated regularly with valsartan 160 mg per day, he had abnormal renal function (the serum level of creatinine was 202 μmol/L). He was on dialysis since the age of 39 years. He denied a history of hearing impairment, but reported myopia. Her uncle (II-3) also exhibited microscopic hematuria and proteinuria with normal renal function, and the renal biopsy, performed at age 24, revealed focal proliferative IgA nephropathy based on immunofluorescence microscopy showing 1+ glomerular mesangial IgA deposits (weak staining) with trace IgG. Regrettably, ultrastructural findings were not documented. Thereafter, he did not accept any antiproteinuric treatment. At age 32, he initiated dialysis therapy because of ESRD. A history of any extrarenal manifestations was not available. Using urinary dipstick testing, blood in a freshly voided urine specimen was detected in the proband's grandmother (I-1), whereas her grandfather (I-2) had a negative result. Unfortunately, I-1 refused further laboratory investigation. The proband, her father and uncle did not undergo an audiogram or an ophthalmological examination. The family was referred to our hospital for diagnosis or exception of Alport syndrome. After obtaining informed consent, a skin sample was obtained from the patient's father for immunostaining for the a1 and a5 chains of type IV collagen and analyzing COL4A5 cDNA. In addition, the patient, her father and her paternal relatives gave blood samples for extracting genomic DNA.

### Immunofluorescence Staining of Skin Specimens

The indirect immunofluorescence method used for analysis of distribution of a (IV) chains has been described previously (2, 13). Murine monoclonal antibodies that recognized the NC1 domains of a1 (IV) and a5 (IV) chains (Wieslab, Lund, Sweden) were used.

### COL4A5 Gene Analysis

According to our previous report (14), the entire coding region of COL4A5 mRNA from the patient's father cultured skin fibroblasts was analyzed by using reverse-transcription polymerase chain reaction (RT-PCR) and direct sequencing. To examine whether the alternatively COL4A5 mRNA transcripts existed in cultured skin fibroblasts, a 232 bp fragment of COL4A5 cDNA, including exons 41A, 41B, and partial sequences of exons 41 and 42, was amplified by PCR using a pair of primers (F: 5′-GCCTTCCAGGTCCAAAGG-3′, R: 5′-CCTGGATTTCCCTTCTCTCC-3′). Then PCR products were separated on an ABI 373 automated sequencer (PerkinElmer) and analyzed by GeneScan software (version 9.1, Metairie, LA). The primers were made based on the published sequence (NM_0033380), and the forward primer was 6-carboxyfluorescein (FAM) labeled. Cultured skin fibroblasts from the patient's father and two healthy male volunteers were used. Genomic DNA of the patient, her father, uncle, grandmother, grandfather as well as at least 100 chromosomes in a control Han Chinese population were screened for this variant using PCR and direct sequencing. A set of primers for exon 41A (F: 5′-tttttgttaatgatgacat-3′, R: 5′-cagaaacactgggttctaca-3′), designed through Primer 3.0 online, was derived from flanking sequences according to the published sequence.

## Results

**Figure 2:**
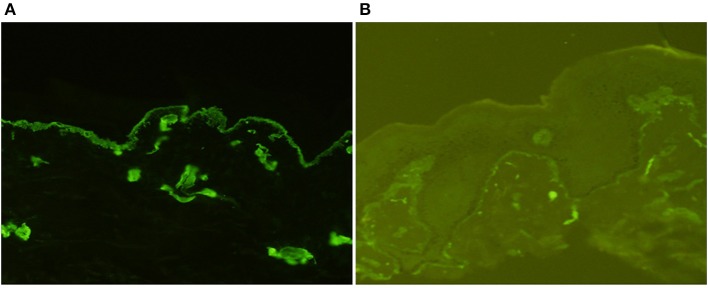
Indirect immunofluorescence with monoclonal antibodies against a1 (IV) and a5 (IV) chains in the epidermal basement membrane from II-1 (×200). There was diffuse linear binding of anti-a1 (IV) antibody to the epidermal basement membrane **(A)**, whereas there was no binding of anti-a5 (IV) antibody to the epidermal basement membrane **(B)**.

**Figure 3:**
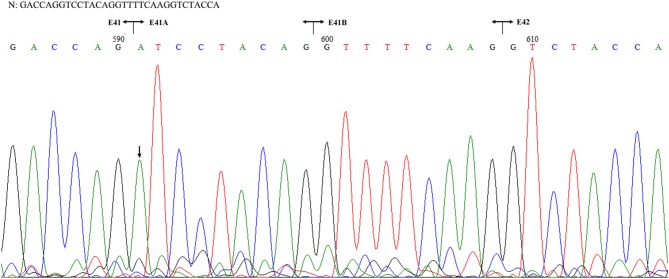
Sequencing of the COL4A5 cDNA isolated from II-1 cultured skin fibroblast. N: normal sequence. The junctions of exons 41, 41A, 41B, and 42 are shown by the vertical line, respectively. The arrow indicated the position of G to A substitution.

**Figure 4:**
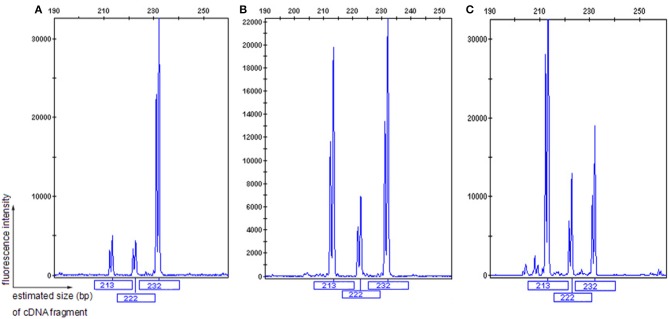
GeneScan analysis of a 232 bp fragment of COL4A5 cDNA, encompassing exons 41A, 41B, and partial sequences of exons 41 and 42. The vertical line (ordinate) shows the fluorescence intensity, and the number beneath each peak is the estimated size (bp) of cDNA fragments. Both II-1 **(A)** and 2 normal males **(B,C)** had three COL4A5 mRNA isoforms in the skin, with a diversity of expression.

**Figure 5:**
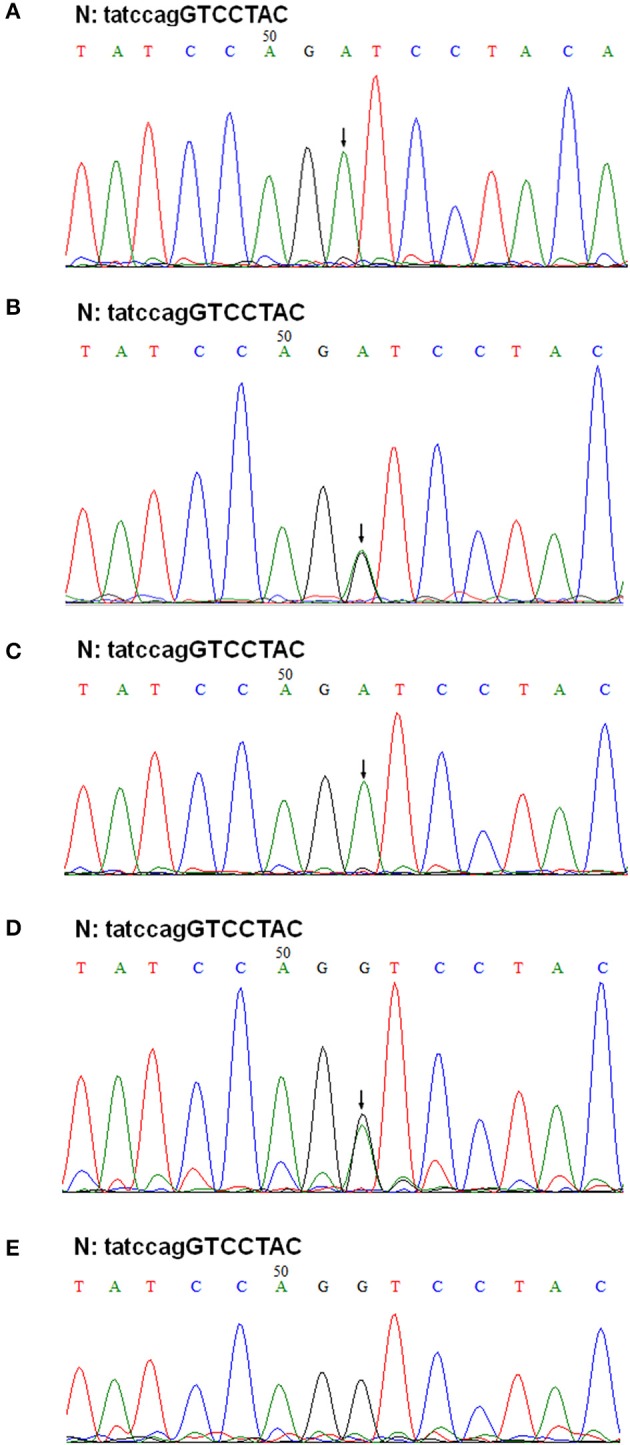
Genomic DNA sequencing of exon 41A in the COL4A5 gene. Sequencing of PCR amplified products of exon 41A from II-1, III-1, II-2, I-1, and I-2 are shown in **(A–E)**, respectively. N, normal sequence. Intron and exon sequences are depicted by lower and capital case letters, respectively. The arrows indicated the position of G to A substitution.

The patient's father (II-1) was found to have positive staining for the a1(IV) chain and negative staining for the a5(IV) chain in EBM (Figure 2). Sequencing analysis of COL4A5 cDNA demonstrated that the dominant transcript containing exons 41A and 41B was present in the cultured skin fibroblasts from the patient's father (Figure 3). A single base change at position 3791 (NM_0033380, namely, the first base of exon 41A) with a substitution of G >A was revealed (Figure 3). The nucleotide substitution converted a GGT codon to a GAT codon at position 1264 (Gly1264Asp). Sequencing the products of two other independent RT-PCR assays showed identical results. The results obtained by the GeneScan analysis of the patient's father and two normal males are shown in Figure 4 and Table 1. There were three COL4A5 mRNA transcripts in the cultured skin fibroblast: the alternative splice product without exons 41A and 41B, the alternative splice product with exon 41B and the alternative splice product with exons 41A and 41B. Variation could be seen in the amounts of amplified isoforms. The variant identified in COL4A5 cDNA was confirmed at the genomic level by amplification and sequencing of the corresponding region in exon 41A. The patient's father and uncle were hemizygous for the mutant allele (Figures 5A,C), and the patient and her grandmother were heterozygous with one normal allele and one mutated allele (Figures 5B,D). I-2's DNA (Figure 5E) and control chromosomes were negative for the variant c. 3791 G > A screen. The variant c.3791G > A (p. Gly1264Asp) in COL4A5 (NM_033380.2) had not been reported in the control database such as NCBI dbSNP build141, the 1,000 Genomes Project, ESP6500, ExAC and gnomAD. The annotation tool ANNOVAR predicted that this variant would be damaging (Table 2). Neither HGMD nor ClinVar documented this variant.

**Table 1:** GeneScan analysis of COL4A5 transcription in the skin^*^.

	**The expression of COL4A5 mRNA isoforms**		
	**Isoform 1 (size: 232 bp) (%)**	**Isoform 2 (size: 222 bp) (%)**	**Isoform 3 (size: 213 bp) (%)**
II-2	77.5	11.8	10.7
One normal male	54.9	14.8	39.3
Another normal male	25.4	17.3	57.3

* *The amount of amplified transcript was indicated by the percentage of the corresponding peak area to total peak areas presenting in the same sample. Isoform 1: the alternative splice product with exons 41A and 41B; Isoform 2: the alternative splice product with exon 41B; Isoform 3: the alternative splice product without exons 41A and 41B*.

**Table 2:** The variant c.3791G > A (p. Gly1264Asp) in COL4A5 (NM_033380.2) annotated by ANNOVAR.

**Gene**	**COL4A5 (NM_033380.2)**
Position	ChrX(hg19): 107913458
Single nucleotide variant	c.3791G>A, p.Gly1264Asp
SIFT	Deleterious
Polyphen2_HVAR	Probably damaging
LRT	Deleterious
MutationTaster	Deleterious
FATHMM	Deleterious
PROVEAN	Deleterious
CADD	Deleterious
M-CAP	Deleterious
MutationAssessor	Conserved
GERP++_RS	Conserved
REVEL	Conserved
phastCons-vertebrate	Conserved
PhyloP Vertebrate	Conserved

## Discussion

Previous studies have reported that abnormal immunostaining in the EBM for the a5(IV) chain was a diagnostic criterion for the disease (2, 15–18). Therefore, even without the ultrastructural findings on kidney biopsy of the proband's father and uncle, the reported family with early onset of glomerular hematuria in the proband; a paternal family history of hematuria, proteinuria and chronic renal dysfunction; and negative a5(IV) chain staining in the EBM of the proband's father could be clinically diagnosed with X-linked AS. The subsequent genetic analysis showed that the proband's father had a novel variant c.3791G > A [p. (Gly1264Asp)] in COL4A5 exon 41A detected at the mRNA and genomic DNA levels, and the variant segregated with disease in the family. According to American College of Medical Genetics and Genomics guidelines (19), this variant was classed as likely pathogenic (PM1+PM2+PP1+PP2+PP3+PP4). Given the evidence that this is a family with X-linked AS, this variant was considered clinically pathogenic. In other words, the development of renal disease in the reported family could be sufficiently explained by the identified variant. Unfortunately, the lack of availability of complete clinical data has impeded further study of the relationship between the variant and deafness or ocular abnormalities.

According to the onset age of ESRD, AS is classified into juvenile (<30 years) and adult (>30 years) forms. Males with X-linked juvenile AS often have extrarenal features, whereas those with adult AS only have sensorineural hearing loss. Their disease severity is influenced by mutation type (7–9). For example, studies of populations in Europe and the United States showed that the risk of developing ESRD at juvenile age is smaller in males with missense mutations than in those with other types of mutations. However, regarding the correlation between the position of glycine substitution and the age of onset of ESRD, clear conclusions have not been drawn (8, 9). As discovered here, although the exact timing of ESRD in the proband's uncle (II-3) was unavailable, according to his age of onset of dialysis, we speculated that the variant c.3791G > A [p. (Gly1264Asp)] may be associated with X-linked adult AS. As far as intrafamilial heterogeneity of the rate of progression to ESRD in the proband's father and uncle was concerned, one possible explanation is the efficiency of angiotensin receptor blockade: there is evidence of nephroprotective effect of renin–angiotensin–aldosterone system inhibition in AS and proteinuric nephropathies (3, 20, 21). Another possible explanation is that the proband's uncle (II-3) suffered from AS accompanied by IgA nephropathy. There were two reports of IgA nephropathy in patients with AS (22, 23). The explanation for the female proband only with microscopic hematuria is probably due to X-chromosomal inactivation in renal tissue rather than the genotype (10, 24).

One study reported a substitution of G > A at position 3790 in COL4A5 exon 41 (NM_0033380. the last nucleotide of exon 41) resulted in skipping of exon 41 (25), whereas functional interpretation of the nucleotide substitution was wrongly documented as p. (Gly1264Ser) in HGMD. Therefore, a substitution of G > A at position 3791 in exon 41A was identified at the mRNA and genomic DNA levels in the proband's father. In addition, three distinct COL4A5 transcripts were detected in the cultured skin fibroblasts, and we speculated that the variant c.3791G > A might result in aberrant splicing patterns. However, the evidence that three COL4A5 transcripts were also detected in the cultured skin fibroblasts of two healthy male volunteers ruled out the possibility of the variant c.3791G > A producing a new splice site. The reason for the diverse expression of three COL4A5 transcripts in skin tissue remains to be elucidated.

In summary, this is the first report on a glycine substitution in COL4A5 exon 41A in a family with intrafamilial heterogeneity of the rate of progression to ESRD in male patients, which extends the phenotypic and mutational spectrum of the disease. In addition, skin tissue has three distinct COL4A5 transcripts with a diversity of expression.

## Data Availability Statement

All datasets generated for this study are included in the article/supplementary material.

## Ethics Statement

The studies involving human participants were reviewed and approved by the Ethical Committee of Peking University First Hospital. Written informed consent was obtained from individual participants or the minors' legal guardian for the publication of any potentially identifiable images or data included in this article.

## Author Contributions

FW designed the study, interpreted the results, and drafted the manuscript. JD contributed to supervision, writing–review and editing, and project administration. DZ and XL performed the experiments. All authors contributed to manuscript revision, read, and approved the submitted manuscript.

### Conflict of Interest

The authors declare that the research was conducted in the absence of any commercial or financial relationships that could be construed as a potential conflict of interest.
